# Depression with comorbid borderline personality disorder - could ketamine be a treatment catalyst?

**DOI:** 10.3389/fpsyt.2024.1398859

**Published:** 2024-04-29

**Authors:** Magdalena Więdłocha, Piotr Marcinowicz, Jan Komarnicki, Małgorzata Tobiaszewska, Weronika Dębowska, Marta Dębowska, Agata Szulc

**Affiliations:** ^1^ Department of Psychiatry, Faculty of Health Sciences, Medical University of Warsaw, Pruszkow, Masovian, Poland; ^2^ KeyClinic, Warsaw, Poland; ^3^ Leszek Giec Upper-Silesian Medical Centre of the Medical University of Silesia, Katowice, Poland; ^4^ Medical University of Warsaw, Warsaw, Masovian, Poland; ^5^ MindHealth, Warsaw, Poland

**Keywords:** ketamine, esketamine, depression, treatment resistant depression (TRD), borderline personality disorder, ketamine-assisted psychotherapy (KAT)

## Abstract

Borderline personality disorder (BPD) is diagnosed in 10-30% of patients with major depressive disorder (MDD), and the frequency of MDD among individuals with BPD reaches over 80%. The comorbidity of MDD and BPD is associated with more severe depressive symptoms and functional impairment, higher risk of treatment resistance and increased suicidality. The effectiveness of ketamine usage in treatment resistant depression (TRD) has been demonstrated in numerous studies. In most of these studies, individuals with BPD were not excluded, thus given the high co-occurrence of these disorders, it is possible that the beneficial effects of ketamine also extend to the subpopulation with comorbid TRD and BPD. However, no protocols were developed that would account for comorbidity. Moreover, psychotherapeutic interventions, which may be crucial for achieving a lasting therapeutic effect in TRD and BPD comorbidity, were not included. In the article, we discuss the results of a small number of existing studies and case reports on the use of ketamine in depressive disorders with comorbid BPD. We elucidate how, at the molecular and brain network levels, ketamine can impact the neurobiology and symptoms of BPD. Furthermore, we explore whether ketamine-induced neuroplasticity, augmented by psychotherapy, could be of use in alleviating core BPD-related symptoms such as emotional dysregulation, self-identity disturbances and self-harming behaviors. We also discuss the potential of ketamine-assisted psychotherapy (KAP) in BPD treatment. As there is no standard approach to the application of ketamine or KAP in individuals with comorbid TRD and BPD, we consider further research in the field as imperative. The priorities should include development of dedicated protocols, distinguishing subpopulations that may benefit most from such treatment and investigating factors that may influence its effectiveness and safety.

## Introduction

Borderline personality disorder (BPD) is diagnosed in 10-30% patients with major depressive disorder (MDD), whereas the incidence of MDD in BPD individuals ranges from 71% to 83% (1–3). Comorbidity of BPD and MDD negatively affects prognosis of both disorders and is associated with more severe depressive symptoms and functional impairment, delayed time to remission and shorter time to relapse (4, 5). Moreover, available treatment options such as antidepressants, electroconvulsive therapy, and psychotherapy are far less effective in such individuals (6–8). In this article we elucidate how, at the molecular and brain network levels, ketamine can impact the neurobiology and symptoms of BPD. We also discuss the results of existing studies and case reports on the use of ketamine/esketamine in BPD or depressive disorders with comorbid BPD. Furthermore, we explore whether ketamine-induced, psychotherapy-augmented neuroplasticity, augmented by psychotherapy, could prove effective in alleviating core BPD-related symptoms. Moreover, we discuss the potential of ketamine-assisted psychotherapy (KAP) in MDD with comorbid BPD.

## Clinical outline

According to International Classification of Diseases 11th Revision (ICD-11) borderline personality is a pattern specifier used in combination with a personality disorder category or a personality difficulty. It may be applied to individuals whose personality disturbance is characterized by a pervasive instability of interpersonal relationships, self-image, affects and marked impulsivity (9). Subjects with BPD experience profound mood disturbances, persistent negative affect and excessive emotional reactions especially in response to social rejection and abandonment (10, 11). Both MDD and BPD highly correlate with non-suicidal self-injuries (NSSI) (12). NSSI is common in BPD patients (50-80% of cases) and approximately 40% of patients committed more than 50 self-mutilations (13). It is estimated that 40 to 85% of BPD individuals attempt suicide, usually multiple times, and up to 10% die as a result (13, 14). Soloff et al. found that comorbidity of BPD with MDD increases the number and severity of suicide attempts (15). A recent study supported findings that comorbid BPD plays crucial role as a risk factor for suicide attempts in depression (16).

Other core features of BPD include impulsivity, emotional dysregulation and disturbed self-identity (17–19). Impulsive behavior in BPD is closely linked to emotional suffering and low distress tolerance (20). Emotional dysregulation is related to heightened negative affect, sensitivity, low self-awareness and deficits in applying regulation strategies (18). Instead of adaptive regulation, maladaptive coping mechanisms are present. These include ruminations, NSSI, impulsive suicidal behaviors and substance abuse (11). Soloff et al. observed that negative affectivity is linked with clinical severity of suicide attempts and reduced inhibitory control (21). A high percentage of patients exhibit stress-related dissociative experiences such as derealization and depersonalization, which, along with the desire to reduce emotional tension, are the main driving factors for self-harm in BPD (20).

Self-identity disturbances in BPD manifest as an inconsistent, non-integrated sense of self and unstable, usually negative self-esteem (20). Individuals with BPD experience high levels of self-criticism, low self-compassion, strongly impaired self-reflection and disoriented life narratives (19, 22). These disturbances result in distrust in their own judgment and long-term difficulties with self- and goal-oriented behavior (20). Moreover, high self-criticism and low self-compassion are related to NSSI (23).

In patients with MDD and BPD, the prevalence of post-traumatic stress disorder (PTSD) is significantly higher than in patients without BPD diagnosis (24). It is estimated that 22-24% of subjects with primary diagnosis of PTSD have comorbid BPD, whereas the prevalence of PTSD in BPD population ranges from 33 to 79% (25, 26). Thus, the comorbidity of BPD and PTSD, as well as BPD with PTSD and MDD seems to be relatively frequent. It is perhaps unsurprising given that BPD is considered a potential risk factor for PTSD (24). In comparison with single-disorder groups, these patients often experienced greater exposure to trauma and more severe mood instability (27). Traumatic or disturbed early relationship experiences may result in insecure attachment patterns and impaired emotional processing (28). It is worth mentioning that complex PTSD (cPTSD), a diagnostic category added recently to ICD-11, in addition to PTSD symptoms, is characterized by disturbances in self-organization, which are conceptualized similarly to BPD symptoms (9).

## Potential neurobiological background of BPD symptoms

In BPD brain dysfunction centers around hypoactive anterior cingulate cortex (ACC), hyperactive amygdala and insula, as well as functional dysconnectivity within and between large brain networks (11). Although recent meta-analysis showed no consistent pattern of alterations in brain activity, it reported a dysfunction of amygdala and ACC during processing of emotional stimuli (29). Goldstein et al. found that BPD subjects, when exposed to repeated negative stimuli, exhibit amplified amygdala response. This evidences impaired amygdala habituation (30). Extensive response to negatively valenced information is associated with higher anxiety, aggression and affective instability levels (11). Hyperresponsiveness of amygdala may prompt individuals to excessively process negative affective stimuli. For BPD subjects, painful stimuli were proven to normalize stress levels and amygdala activity, which may explain frequent NSSI (31, 32).

Baczkowski et al. demonstrated that in BPD, an increase in connectivity resulting from performing emotional regulation tasks does not occur in regions essential for effortful emotional regulation, such as prefrontal cortex (PFC). As a result, cognitive control, which enables reinterpretation of meaning of emotional stimuli, is impaired (33). Frontolimbic dysconnectivity hypothesis, which includes deficient top-down control and enhanced bottom-up regulation, explains the neural mechanism of affective instability in BPD, as well as preoccupation with negative ideation in MDD (11, 34). Reduced top-down regulatory activity in brain regions supporting cognitive control such as dorsolateral PFC (dlPFC) and dorsal ACC (dACC) may result in the inability to suppress distracting emotional influences (35). On the other hand, abnormal bottom-up regulation is linked with increased amygdala activity. It results in excessive responses to emotional stimuli that dysregulate cognitive control (34).

A growing body of evidence based on resting state functional magnetic resonance (rs-fMRI), supports the presence of alterations in functional network connectivity in BPD. Aguilar-Ortiz et al. showed failures in deactivation in key regions of default mode network (DMN), such as medial frontal cortex and the precuneus (36). Activity within DMN is related to internally directed, self-referential processes and ruminations (37). O’Neil et al. reported increased connectivity between precuneus and frontal regions, which are responsible for processing of self-referential thoughts and information (38). Ruminative thinking triggered by negative affect influences severity of BPD symptoms (39). Van Schie et al. indicated that in BPD individuals, altered activity of temporolimbic areas and precuneus leads to focusing on negative feedback which maintains their negative self-esteem (40). Heightened sensitivity to social exclusion may be significantly associated with precuneus and insula activation (41). Abnormal activation of the insula, one of the key salience network (SN) nodes, during affective and pain regulation is believed to be one of neural mechanisms underlying NSSI in BPD patients (42). In BPD, hyperconnectivity within SN nodes (amygdala and insula with dACC) is associated with emotional hypersensitivity, whereas reduced connectivity between SN and frontoparietal regions of central executive network (CEN) contributes to impaired control over emotional reactions (43).

Among neurobiological alterations present in BPD, opioid neurotransmission disturbances are also of interest. Low basal opioid concentration may manifest as chronic dysphoria and a lack of sense of wellbeing. Low opioid levels along with compensatory higher sensitivity of μ-opioid receptors may explain repetitive NSSI as a behavior which leads to increase in opioid neurotransmission (44). Adverse experiences, such as childhood abuse, common in BPD, are thought to result in modulation of the opioid system (45). Importantly, intrapsychic pain, same as the physical, is regulated by opioids and the neural network comprising e.g. ACC, insula, amygdala, hypothalamus and nucleus accumbens (46). Opioid disturbances in BPD can contribute to emotional suffering related to social rejection or exclusion manifesting in self-harm and suicide (47).

## Current BPD treatment outlook

There is no approved pharmacological treatment for BPD (20). Additionally, meta-analyses have shown that no pharmacotherapy appears to be effective for the overall severity of BPD symptoms (48, 49). However, some agents prove to be beneficial in several types of BPD symptoms, thus a symptom-targeted pharmacotherapy is a common strategy in clinical practice (50). Selective serotonin (SSRIs) and serotonin and norepinephrine (SNRI) reuptake inhibitors may be beneficial in reducing impulsivity, affective lability, irritability and somatic symptoms, although there is no conclusive evidence that they may contribute to consistent reduction of the severity of BPD (51, 52). According to American Psychiatric Association (APA) guidelines, SSRI or SNRI should be a first-line pharmacological treatment of affective dysregulation and impulsive-behavioral dyscontrol symptoms in BPD (53). On the other hand, in a more recent review, Bohus et al. conclude, that there is no sufficient evidence to support SSRI use in the treatment of BPD psychopathology, unless antidepressant effect is required (20). Low-certainty, limited evidence suggests that anticonvulsants such as valproate, lamotrigine and topiramate can be beneficial in anger, aggression, and affective lability associated with BPD (51). However, as APA guidelines indicate, mood stabilizers (lithium, valproate or carbamazepine) may be considered as a second-line or adjunctive treatment of symptoms within the above domains (53). Second generation antipsychotics have been reported to reduce anger, affective instability, impulsivity, paranoid ideation, dissociative symptoms and anxiety in BPD (52). APA guidelines recommend those particularly in treatment of cognitive-perceptual BPD symptoms, whereas The National Institute for Health and Care Excellence (NICE) guidelines state that antipsychotics can be considered only as a crisis treatment, prescribed for no longer than 1 week (54). A recently published comparative effectiveness research study, indicated that among all pharmacotherapies employed in BPD patients, only the treatment with attention deficit hyperactivity disorder medication was associated with a reduced risk of suicidal behaviors (55). Some authors suggest that therapy of BPD needs to be prioritized when BPD and depression co-occur (1). It seems more accurate however, that non-BPD disorder (i.e. MDD) should be managed in parallel with BPD-oriented psychotherapy (20).

Among BPD-specific psychotherapies, dialectical behavior therapy (DBT) and mentalization-based treatment (MBT) have been studied most extensively. Transference-focused psychotherapy (TFP) and schema-focused therapy (SFT) are also established psychotherapeutic strategies for BPD (56). DBT focuses on symptoms of emotional dysregulation, MBT – difficulties in identifying oneself and others mental states, TFP – unintegrated, undifferentiated images and representations of oneself and others, often following early-experienced trauma, while SFT - dysfunctional life schemas and thinking patterns (20). BPD-specific approaches were shown to support improvements in BPD symptoms and psychological well-being, but their effectiveness is reported to be moderate. Additionally, they do not fulfil the need for rapid symptom reduction, have limited accessibility and high dropout rate (57, 58). A recent review of 28 studies of various modalities psychotherapy in BPD (with DBT as the most frequent) indicated that approximately half of the patients did not respond to treatment and over a quarter of patients dropped out (56). A meta-analysis of DBT studies regarding its impact on suicidality revealed reduced self-directed violence and frequency of crisis services interventions with no significant improvement in suicidal thoughts (59). A recent Cochrane review of psychotherapies applied in BPD found no improvement in interpersonal and psychosocial functioning, fear of abandonment, affective instability and feeling of emptiness at 6 to 12 months after the end of treatment (49).

## Antidepressant and antisuicidal efficacy of ketamine

Ketamine administration in MDD and treatment resistant depression (TRD) is widely researched, with its efficacy evidenced in numerous double-blind, randomized clinical trials (RCT) (60–67). It is regarded as fast-acting antidepressant (68–70). Kryst et al. have shown that a single infusion may result in a significant antidepressive effect lasting for up to 7 days, which can be sustained by repeated infusions (70). The vast majority of RCTs of ketamine in MDD did not exclude patients with comorbid BPD (60–67). In a midazolam-controlled study on MDD individuals with significant suicidal ideation, 28% of participants met the diagnostic criteria for BPD, with ketamine proving to be superior in reduction of depressive symptoms and suicidal ideation within 24 hours after the single infusion. The authors reported that clinical improvement was maintained for up to 6 weeks (71). Given the high co-occurrence of MDD and BPD, it is possible that the beneficial effects of ketamine can also extend to the subpopulation with BPD. However, no protocols were developed that would account for this comorbidity. Additionally, many of the esketamine randomized clinical trials excluded individuals with BPD (72–75). Notwithstanding, real-world study of esketamine in TRD including 15% of individuals with comorbid personality disorders, indicated significant reduction of depressive symptoms and suicidal thoughts (76). Three months after beginning of treatment, clinical response and remission rates were high - 64,2% and 40,6%, respectively. Moreover, no differences in efficacy of esketamine were found among patients with and without comorbid personality disorders.

Both ketamine and esketamine are proven to rapidly decrease suicidal thoughts. Chen et al. assessed the antisuicidal effect of ketamine as ‘large’ or ‘medium-large’ (after 4-6 and 24 hours after infusion, respectively), whereas the effect of intranasal esketamine was reported as ‘small-medium’ (77). Ketamine-induced decrease in suicidal thoughts may be partially independent of the improvement in depressive symptoms (71). Lengvenyte et al. suggested that ketamine may be particularly useful in patients with stress-induced suicidal ideation, which is common in BPD (47).

## Ketamine’s mechanisms of antidepressant action

Ketamine is a racemic mixture of two enantiomers, esketamine and arketamine (78). It is a nonselective, noncompetitive N-methyl-D-aspartate receptor (NMDAR) antagonist, which binds to the phencyclidine site of this receptor (78). Importantly, ketamine preferentially blocks NMDAR on the inhibitory gamma-aminobutyric acid (GABA) interneurons. This preferential action of ketamine leads to pyramidal cell disinhibition and an increase in overall excitatory glutamatergic neurotransmission, especially the prefrontal cortex and cortico-limbic regions, which are associated with mood regulation (79). Ketamine is hypothesized to inhibit extra-synaptic GluN2B-NMDAR. Their activation results in suppression of protein synthesis. Therefore, the blockade of GluN2B-NMDAR de-suppresses protein synthesis, which may induce antidepressant action via a mechanistic target of rapamycin (mTOR)-dependent pathway (80). However, it seems that blocking NMDAR may not be the main mechanism of ketamine’s therapeutic effect, as studies of other NMDAR antagonists did not show their antidepressant efficacy (68, 81). Meta-analysis of placebo-controlled trials using racemic ketamine or esketamine did not show greater antidepressant efficacy of esketamine, even though esketamine has a 3-4 times greater affinity for NMDAR than arketamine (69). In turn, arketamine, despite its lower affinity for NMDAR showed a greater antidepressant effect in preclinical studies (82, 83). (2R, 6R)-hydroxynorketamine, a metabolite of arketamine with low affinity for NMDAR, also showed a rapid antidepressant effect in rodents. It has been proposed that this metabolite might be a key component of ketamine’s antidepressant effectiveness (83). However, it was not confirmed in studies on patients with depression, as higher level of hydroxynorketamine was associated with less significant clinical improvement (84, 85).

The aforementioned prefrontal cortex disinhibition is thought to be associated with an increase in dopaminergic, serotoninergic and noradrenergic transmissions in cortical and subcortical brain regions (79). In the region of lateral habenula, regarded as an ‘anti-reward center’ because of its engagement in negative emotion coding, ketamine inhibits NMDA dependent neuronal bursting activity (86). Subsequently, the downstream monoaminergic reward centers in ventral tegmental area and dorsal raphe nucleus become disinhibited, the reward processing is restored and pleasure perception increases (47).

Ketamine increases activity of α-amino-3-hydroxy-5-methyl-4-isoxazolepropionic acid receptors (AMPAR) which play crucial role in long-term potentiation (LTP). LTP is one of phenomena underlying synaptic plasticity, that results in a persistent strengthening of synapses (87). AMPAR activation leads to the release of the brain derived neurotrophic factor (BDNF) and enhances the availability of its tropomyosin kinase B (TRKB) receptor (87, 88). Neuroplasticity is considered as a key mechanism of ketamine antidepressive action. Meta-analysis on the potential biomarkers of ketamine efficacy indicated that patients who exhibited increased BDNF levels during treatment were more likely to become responders (89).

Furthermore, ketamine and esketamine are thought to share several mechanisms of action with mood stabilizers and act as cellular membrane stabilizers, as well as modulators of neuronal excitability. Acting on GluN2D NMDAR subunits reduces the influx of Ca2+ ions, which leads to restoration of membrane potential which subsequently alters protein translation and availability which finally results in neuroplasticity enhancement (90). Preclinical studies revealed that ketamine and, to a greater extent, esketamine may also inhibit the voltage-gated sodium channels (VGSC) and reduce the influx of Na+, which in turn decreases the excitatory neurotransmission (91). Importantly, this mechanism of action forms a molecular basis of therapeutic effect of several mood stabilizers such as valproate, carbamazepine and lamotrigine (92). On the other hand, ketamine, similarly to lithium, inhibits the glycogen synthase kinase 3β (GSK-3β) pathways (through GSK-3β phosphorylation), which is considered as possible significant mechanism contributing to its antidepressant and neuroplastic effect (93). As mood stabilizers are reported to be, to a certain extent, effective in reducing impulsivity, aggression and anger in BPD, the above molecular effects of ketamine may also prove to be advantageous in treatment of depression with comorbid BPD. Interestingly, McIntyre et al. indicated that ketamine may be effective in treatment-resistant MDD or bipolar disorder with mixed features such as anxiety, irritability and agitation (94).

Influence that ketamine exerts on the opioid system may prove beneficial in BPD, in which opioid neurotransmission seems to be disturbed. Ketamine as an agonist of opioid receptors increases basal opioid levels (83). Research indicates that blocking the opioid receptors with naltrexone reduces both antidepressant and antisuicidal effects (95). Moreover, it is suggested that dynorphins, as an endogenic agonist of κ-opioid receptors, may mediate emotional pain, dysphoria and promote self-harm behaviors (96). Ketamine is thought to cause down-regulation of κ-opioid receptors and resolve imbalance between ‘hedonic’ μ- and ‘dysphoric’ κ-opioid receptors activity (97). We speculate that ketamine modulatory effect on opioid neurotransmission may contribute to reduction in negative affect and autodestructive tendencies in BPD individuals.

## Ketamine-induced alterations in brain activity

Numerous studies indicate that remitters treated with ketamine exhibit normalization in intra- and inter-network functional connectivity (67, 98, 99). ACC-related circuit modulation is thought to be crucial in ketamine antidepressant and antisuicidal action (100). Alexander speculates that ketamine acute effects on subgenual ACC reflect in shutting down emotional pain network and alleviating affective pain, whereas sustained effects on neuroplastic modulation in DMN contribute to resolving of ruminative thinking patterns (97). Similarly to serotoninergic psychedelics, ketamine has been shown to acutely disintegrate functional connectivity in DMN and decrease activity within this network (101, 102).

Evans et al. indicated normalization of the interaction between DMN and SN in MDD individuals after ketamine infusion (103). Ketamine also increases connectivity between DMN and CEN nodes (104–108). It could prove beneficial for both MDD and BPD patients as such increase facilitates shifting attention from internal, self-referential thought processes towards external, goal-directed tasks (109). Vasavada et al. indicated that repeated ketamine administrations lead to increased top-down control of emotional processes and restored top-down regulation of ventral limbic structures (110). Sterpenich et al. have reported that ketamine application resulted in decreased amygdala, insula and dACC responses to negative stimuli during an emotional recognition task (111). Normalization of these SN nodes overactivity is thought to play an important role in the antidepressant effect (43, 112).

Ketamine is also reported to alleviate stress-related symptoms by enhancing neuroplasticity particularly in medial PFC (mPFC). Norbury et al. revealed that post-traumatic stress disorder (PTSD) symptoms improvement in ketamine group was associated with increased prefrontal top-down inhibition of amygdala in response to social signs of a threat. Moreover, individuals with lower baseline mPFC inhibition of amygdala showed greater clinical improvement as a result of ketamine treatment (113). The effect could also prove beneficial in BPD treatment, given that PTSD and BPD both exhibit reduced activation of executive-related frontal regions and hyperactivation of the emotion-related limbic regions.

Frontostriatal and interlimbic connectivity normalization caused by ketamine is thought to facilitate regaining cognitive control over emotional activity (101). It may prove significant for patients with comorbid depression and BPD who exhibit abnormalities in top-down and bottom-up processing. We speculate that ketamine impact on intra- and inter-network connectivity induces long-lasting cognitive and psychological flexibility, which in turn contributes to improvement in BPD-related negative self-schema and disturbed social cognition. Enhancement of neuroplasticity between limbic regions and networks essential for emotional regulation, self-awareness, goal-oriented and social behaviors may meaningfully impact treatment of TRD with comorbid BPD.

On the other hand, it was also reported that serial ketamine infusions result in significant decrease in activation of brain regions associated with response inhibition and inhibitory control network, which is related to improvement in depressive symptoms (114). Such normalization, while beneficial in TRD, may result in increased impulsivity and self-harming behaviors in comorbid BPD.

Stone et al. reported reduced activation in the left superior temporal cortex after ketamine infusion, which is associated with impaired self-monitoring (115). Hyperactive self-monitoring is considered to be a part of depression mindset, thus its reduction may be beneficial for MDD patients (116). However, in BPD individuals reduced ability to self-monitor may disrupt already low emotional awareness.

## Ketamine/esketamine trials and case studies in BPD

Danyan et al. evaluated the therapeutic effect of ketamine (4 intravenous infusions in 2 weeks, 0,5-0,75mg/kg) in TRD patients with and without comorbid BPD. Both groups showed comparable improvement in depressive and anxiety symptoms, as well as in intensity of suicidal ideations. Reduction in depressive and BPD symptoms (measured with Borderline Symptom List, BSL-23) and positive correlation between these improvements was indicated. The antidepressant effect of ketamine was more pronounced in patients with more severe baseline suicidal ideation. Moreover, improvements in social, family and work functionality scores were observed. Dissociative symptoms were mild and transient in both groups. Relevant limitations of the study included retrospective and open-label design and short (1 week) follow-up after final infusion (117).

In an open-label study Chen et al. explored the effectiveness and safety of single intravenous infusion (0,5mg/kg) in MDD individuals with or without elevated BPD features. Improvements in depressive symptoms as well as suicidal ideation were significant and comparable in both groups within 3 and 24h after infusion. In group encompassing MDD subjects with BPD features, the response after 14 days was of greater magnitude. Dissociative symptoms were mild, but more pronounced in BPD group 24h after infusion. Brief Psychiatric Rating Scale (BPRS) scores, reflecting the severity of psychotic symptoms, were very low at all times. It must be noted, however, that the study was not specifically focused on BPD and the groups were differentiated post-hoc (118).

A double blind, randomized, midazolam-controlled pilot study tested the effects of single ketamine infusion (0,5mg/kg) in a small sample of BPD individuals. It revealed no significant changes in suicidal ideation, depression, anxiety or BPD symptoms. A greater decrease in suicidality and depressive symptoms in ketamine group was found, but it was not statistically significant. However, the study indicated improvement in socio-occupational functioning in the ketamine group. Ketamine was well tolerated, no serious adverse events occurred. It is worth noting though, that two participants of the ketamine group experienced acute distress and suicidal ideations in 4th week after infusion - one was discharged after overnight evaluation and the other received further ketamine infusions as a part of inpatient treatment (119).

Nandan et al. published a case report of an 27-year old female with TRD and BPD, hospitalized after a suicide attempt. After initial stabilization in inpatient setting, intranasal esketamine treatment was started in the outpatient setting in conjunction with citalopram and buspirone. Initial esketamine dose equaled 56 mg administered twice a week in four weeks timespan, followed by 56 mg administered once per week, which was further increased to 84 mg once per week. Authors reported significant improvement in depressive symptoms and suicidality, as well as in core BPD symptoms within 4-5 weeks. Esketamine treatment was continued for the next two years with significant improvement observed in depressive symptoms, impulsivity, affective instability and psycho-social functioning. Frequency of self-harm attempts decreased. Nandan et al. reported patient’s full compliance with treatment plan, with it being poor during previous therapies. Notably, the authors indicated the importance of maintenance treatment - when esketamine administration was omitted (due to the unavailability of medication), resurgence of affective instability and self-harm attempts occurred (120).

Another case report refers to 22-year old female with MDD, social phobia, BPD and frequent past NSSI. After two ketamine infusions (0,5mg/kg) during hospitalization, robust improvement in depressive symptoms, suicidal ideation, social functioning, emotional and behavioral dysregulation was observed. Subsequently, the treatment was continued in outpatient setting. During the last follow-up, half a year after first infusion, reduction in depressive and BPD symptoms was observed. The patient completed a 3-month inpatient DBT treatment during this time. Authors speculated that ketamine modulatory effect on neuroplasticity contributed substantially to the satisfactory result of DBT that followed (121).

Galuszko-Węgielnik et al. presented a case report of 26-year old female with BPD and bipolar treatment resistant depression, who was planned to receive 8 intravenous infusions of ketamine (0,5mg/kg). The patient experienced severe dissociative symptoms as a consequence of infusions and the third one was followed by increased suicidal ideation, impulsive behavior and NSSI. No improvement in depression was observed, therefore ketamine treatment was discontinued (122).

Vanicek et al. presented a case report of a 20-year old female with MDD and BPD, who received 5 intravenous infusions of esketamine (25-50mg) within 2 weeks. Initially, a rapid improvement in depressive symptoms and suicidal ideation was observed, but over the course of treatment disinhibition symptoms occurred. Increased emotional responsivity and decreased cognitive control contributed to an impulsive suicide attempt after fifth ketamine infusion. Due to deterioration of patient’s mental condition, ketamine treatment was discontinued (123).

Research suggests that ketamine/esketamine treatment may be beneficial and safe for BPD or BPD with comorbid MDD patients. On the other hand, reports indicate that acute ketamine effects such as dissociation and altered perception of reality and oneself may increase affective instability and impulsive suicidal behaviors. It is worth noting though, that no psychotherapy or psychedelic integration parallel to ketamine/esketamine administrations have been attempted in any of the discussed trials and case studies except for Rogg et al. (121). The psychedelic effect of ketamine may evoke difficult experiences, therefore psychotherapeutic integration may prove essential for individuals with MDD and BPD during ketamine treatment (124).

## Ketamine-assisted psychotherapy

In a recently published systematic review, KAP application was examined in a range of disorders including MDD, PTSD, substance abuse, obsessive-compulsive disorder, generalized anxiety disorder and neuropathic pain. Most studies were focused on cognitive-behavioral therapy (CBT) and mindfulness-based psychotherapy but some involved motivational enhancement therapy, exposure therapy, existentially oriented psychotherapy and functional analytic psychotherapy. Importantly, in most of the KAP-related studies individuals with comorbid BPD weren’t excluded. Definite conclusions and recommendations were not formulated due to differences in psychotherapeutic approaches and research methodologies. It was evidenced however, that incorporation of psychotherapy throughout the course of ketamine treatment may give rise to and maintain clinical improvement by reducing depression, anxiety and pain (125). Dore et al. proved that with KAP incorporation the higher baseline suicidality levels, the greater decrease in affective symptoms (126). Krupitsky et al. applied KAP in individuals with alcohol use disorder, which resulted in improvements in emotional dysregulation and personality characteristics linked to self-criticism (127). Application of KAP in depression with comorbid BPD has not been explored yet.

Wilkinson et al. proposed that ketamine-induced enhancement of neuroplasticity may open a window of opportunity, where cognitive flexibility and learning potential are increased. Authors suggested that ketamine may increase sensitivity within key brain regions (such as mPFC and hippocampus) and induce neuroplastic changes similar as in the use of CBT. It was shown that responders to ketamine exhibited rapid improvement in cognitive control, with CBT strengthening and maintaining that improvement, which in turn may result in reversal of disrupted information processing and maladaptive behaviors (128). Ketamine may also facilitate emotional learning and improvement of negative self-schema, which is one of the core cognitive aspects of both depression and BPD (125, 129). Moreover, ketamine-induced alteration in DMN activity is thought to enable subsequent revision of mental representations of self (102).

Most research involving application of ketamine in the treatment of mental disorders regards acute ketamine-induced symptoms as side effects, with their severity monitored using dissociative and psychotic symptoms scales (most commonly Clinician-Administered Dissociative States Scale and BPRS) (130). However, several studies point out that the quality of subjective experience during ketamine administration may substantially contribute to the overall therapeutic effect. Sumner et al. proved that a greater antidepressant response to ketamine correlated with higher scores in Alerted States of Consciousness (ASC) questionnaire. The study suggests that the psychedelic experience itself may play a significant role in ketamine’s antidepressant properties (124). Aust et al. underpinned importance of considering subjective quality of ketamine induced psychological effects, indicating that anxiety-related experiences may be linked to the absence of the antidepressant effect (131). Subjective experiences were reported as significantly contributing to the therapeutic effect of ketamine not only in MDD. Mystical experiences were associated with improvement in cocaine and alcohol use disorder (132, 133). Krupitsky et al. pointed out, that in addiction treatment ketamine may provide transformative experiences. After being subjected to KAP patients with heroin use disorder rated their sense of control as significantly more ‘internal’, which resulted in a better outcome in heroin abstinence (134). Research also indicates that the transpersonal experience of ketamine may bring on personal insights and stimulate reframing of beliefs (125). Marguilho et al. suggested that psychedelic-assisted psychotherapy efficacy is most accurately predicted by questionnaires assessing subjective psychedelic experience, which involve ego-dissolution, emotional breakthrough and mystical experiences (102). Dore et al. argue that psychedelic and dissociative experiences are an integral part of KAP and should be supported in a psychotherapeutic context (126).

The influence that ketamine has on restructuring of traumatic memories is another potentially important effect in relation to psychotherapeutic treatment in TRD with comorbid BPD. Given the importance of traumatic experiences in BPD development, the conclusions inferred from studying KAP in PTSD are potentially applicable in BPD. Better access to traumatic memories and extinction of previously paired pain-related memories are among potential processes enabling efficacy of ketamine in PTSD treatment (135). Taking into consideration that ketamine’s molecular and neural mechanisms of action are also involved in memory reconsolidation, Fattore et al. speculated that application of ketamine few hours prior to memory retrieval may trigger a metaplastic cascade. Increased synaptic plasticity and alterations in neural connectivity facilitate destabilization of memories and increases receptiveness to non-pharmacological interventions (136). Although there are concerns regarding increased risk of self-harm and suicidal behavior following trauma-focused treatments in BPD patients, a systematic review of psychotherapeutic approaches for comorbid BPD and PTSD treatment indicated that trauma-focused therapies may reduce both PTSD and BPD symptoms, whereas BPD-specific psychotherapies do not alleviate PTSD symptoms (137). Identifying and tying together past experiences and current symptoms may be helpful in understanding how trauma is reflected in patient’s present problems. Integrating ketamine with evidence-based psychotherapy requires further exploration in populations with comorbid depression, BPD and PTSD. Interestingly, a study on 3,4-methyl​enedioxymethamphetamine (MDMA)-assisted psychotherapy in PTSD revealed that the effect of the intervention extended beyond specific PTSD symptomatology and resulted in long-term personality changes such as increased openness and decreased neuroticism (138).

Researchers also point out positive aspects of pairing ketamine with psychotherapy such as reduction in defensiveness and promoting recollection of emotionally arousing past experiences. Moreover, it is suggested that ketamine’s rapid antidepressant and anxiolytic effects may enhance treatment adherence and engagement in building of the therapeutic alliance (125). This may result in considerable progress in BPD treatment, where compliance is low and drop-out rates are significant.

## Limitations and risks of ketamine treatment in TRD patients with comorbid BPD

Increased emotional sensitivity, as well as cognitive and emotional overload during ketamine treatment may be overwhelming for BPD patients, especially in the absence of a therapeutic process. Dissociative symptoms in BPD individuals with a history of dissociation may be exacerbated after ketamine exposure (119). These may lead to self-harm, deterioration in emotional learning and weak psychotherapy response (139–141). Moreover, psychotic-like experiences may be traumatizing for vulnerable individuals. Similarly, reliving traumatic memories, especially outside of the psychotherapeutic context, may be linked with increased risk of self-harm and suicidal behaviors. Taking into account BPD-related low tolerance of frustration and impulsivity, the risk of suicide may greatly increase in the absence of noticeable, rapid antidepressive effect of ketamine that the patient was expecting.

Additionally, the risk of addiction in BPD patients cannot be ignored. In a review of 70 studies, Trull et al. reported that approximately half of BPD patients exhibit at least one substance use disorder (SUD) (with alcohol being the most common), whereas approximately 25% of individuals with SUD also meet criteria for BPD (142). Notwithstanding, in research involving ketamine/esketamine in MDD no substantial risks related to its use in a controlled medical setting were reported, however no studies were performed with a focus on BPD patients, in which substance abuse is a common symptom of behavioral dysregulation (143). Recently, Chiappini et al. provided preliminary insights of effectiveness and safety of intranasal esketamine among TRD patients with comorbid substance use disorder. Antidepressant effect was significant and no cases of abuse of esketamine were reported. Despite significant methodological limitations, the authors considered esketamine as effective and safe in TRD patients with comorbid SUD (144).

## Mitigating risks and improving results of ketamine treatment

NSSI and suicide risk assessment and management strategies, such as development of safety plan based on DBT interventions, should become integral part of the treatment process. In BPD, psychotherapy remains a first line treatment and its incorporation into ketamine treatment protocols appears to be necessary for patients safety and efficacy improvement. Given that exposure to ketamine may provoke strong emotional reactions and trigger maladaptive defense mechanisms in BPD patients, involvement of experienced therapists is critical. It is suggested that more frequent psychotherapeutic sessions and longer duration of psychotherapy leads to increase in the efficacy of KAP (125).

A realistic goal setting is an important theme during preparation to KAP. Introducing patient to various levels of ketamine action (e.g. neurobiological, psychological) may help setting reasonable expectations. Psychoeducation regarding the procedure may decrease the risk of anxiety occurrence and aid with immersion into the psychedelic experience.

The presence of qualified personnel is required to supervise patients physical safety and assist in navigating psychological distress (125). Additionally, the setting of treatment should facilitate relaxation and help with involvement in the psychedelic experience. Ketamine administration should be followed by psychedelic integration session in order for the patient to understand and accept the experience. Psychedelic integration, although variably defined, involves reflection, validation and making meaning of psychedelic experiences and ideally should lead to incorporation of the insights into everyday life (145).

## Suggested direction of future studies

We recommend controlled trials of ketamine/esketamine treatment and assisted psychotherapy in patients with TRD with comorbid BPD to assess efficacy and safety of various protocols in that population. According to available data, we conclude that TRD patients with comorbid BPD are viable candidates for clinical trials when at least 2 adequate pharmacotherapies and psychotherapy turned out to be ineffective. Research involving patients at high suicide risk (e.g. multiple or recent suicide attempts), with frequent NSSI or severe dissociative symptoms should be performed in inpatient setting, where continuous, intensified medical and psychological care is available. In the course of trials it is vital to research whether the suicide ideations and substance abuse risks constitute a major obstacle in ketamine introduction to treatment strategies. Taking into consideration BPD symptoms persistence and their susceptibility to environmental conditions, trials that would include longer lasting follow-up seem to be of most value. Additionally, it is needed to establish the optimal frequency of ketamine administration and psychotherapeutic sessions, duration of treatment, as well as psychotherapeutic modality used in KAP. Some studies suggest superiority of higher doses of ketamine in KAP, thus it is also important to assess the effects of different dosing in TRD with comorbid BPD (126, 132).

Current state of research suggests that severe personality disorders, including BPD, may constitute contraindication to ketamine treatment. Criteria of personality disorders severity included in ICD-11 and DSM-5 are comparable to Kernberg’s level of personality organization approach based on assessment of presence of psychological defense mechanisms, extent of reality testing the level of identity integration and the control of aggression. According to this model, more frequent use of primitive defense mechanisms to cope with stressors and conflicts, low ability to distinguish intrapsychic from external sources of stimuli, poor sense of self, highly disintegrated identity, inability to understand or accept ordinary social criteria of reality, as well as cognitive and affective inadequacy to the psychosocial situations, are considered as indicators of psychotic level of personality organization, reflecting severe personality disorder (146). In our opinion, TRD individuals with comorbid BPD who exhibit such severity of intrapsychic functioning disturbances, should not be qualified for ketamine treatment or KAP.

As the available research is insufficient to distinguish subpopulations that could benefit the most from the ketamine introduction, further research should focus on psychological and neurobiological predictors of the therapy outcome to distinguish clusters of TRD patients with comorbid BPD. Cluster differentiation could be centered around the efficacy and safety of ketamine/esketamine and KAP application in treating patients exhibiting varying intensity of personality traits typically present in BPD such as emotional lability, anxiousness, separation insecurity, depressivity, impulsivity, risk taking and hostility. Potential impact of severity of suicidal ideations, substance abuse and the presence of common comorbid disorders such as PTSD, cPTSD, SUD, ADHD on the treatment outcome should also be of consideration.

As some reports suggest that ketamine and other psychedelics may affect personality traits, further studies are needed to evaluate the impact KAP has on personality dimensions (127, 138). The quality of subjective experience and psychedelic effect should be considered (measured by, for instance, ASC questionnaire) when evaluating clinical outcomes related to both depressive and BPD-specific symptoms such as suicidal ideation, fear of abandonment and feeling of emptiness.

## Summary

BPD is a common comorbidity of TRD and it negatively affects the course, treatment, outcome and prognosis. Moreover, it was shown that in contrast to behavioral symptoms, BPD core affective dysfunctions persist into later course of disorder (147). Interpersonal stressors are known triggers of an affective dysregulation cascade in BPD, which may result in suicidal ideations and attempts (148). Efficacy of the available pharmacological and psychotherapeutic treatments is not sufficient, thus novel therapeutic approaches are needed. Ketamine, which is evidenced to have significant antidepressant and antisuicidal effect, may become one of those. It should be emphasized though, that in vast majority of ketamine trials in MDD, patients with comorbid BPD were not excluded, yet they were not treated as a distinct group. Therefore, the efficacy and safety of the treatment has not yet been evaluated for that population.

What is more, in MDD trials, as well as in a few studies focused on BPD patients, the administration of ketamine was paired with neither psychotherapy nor psychedelic integration. Taking into account the risk of affective decompensation following ketamine exposure, these processes should form a basis of a treatment strategy. Therapeutic interventions may also help with immersion into the ketamine experience, which subjective quality seems to be important for treatment results. Additionally, enhanced neuroplasticity occurring after ketamine administration may increase cognitive flexibility and emotional learning. This can lead to improved responses to psychotherapy.

On a neurobiological level, ketamine-induced changes seem to refer to alterations reported in BPD and result in revision of mental representations of self, as well as in improvements in cognitive control and emotional regulation. It is worth researching whether ketamine-induced normalization in top-down control and bottom-up regulation processes observed in MDD and PTSD could be applicable in MDD with BPD-related emotion dysregulation.

Considering the above, we emphasize the need for extensive research of efficacy and safety of ketamine treatment with assisted psychotherapy in patients suffering from TRD with comorbid BPD. This is a crucial need and a key direction, especially in the absence of effective pharmacotherapy for BPD.

## Author contributions

MW: Conceptualization, Data curation, Investigation, Project administration, Writing – original draft. PM: Investigation, Writing – original draft, Data curation. JK: Conceptualization, Investigation, Writing – original draft. MM: Writing – original draft. WD: Writing – review & editing. MD: Writing – review & editing. AS: Supervision, Writing – review & editing.
