# Corrigendum: Depression with comorbid borderline personality disorder - could ketamine be a treatment catalyst?

**DOI:** 10.3389/fpsyt.2024.1507504

**Published:** 2024-10-28

**Authors:** Magdalena Więdłocha, Piotr Marcinowicz, Jan Komarnicki, Małgorzata Tobiaszewska, Weronika Dębowska, Marta Dębowska, Agata Szulc

**Affiliations:** ^1^ Department of Psychiatry, Faculty of Health Sciences, Medical University of Warsaw, Pruszkow, Masovian, Poland; ^2^ KeyClinic, Warsaw, Poland; ^3^ Leszek Giec Upper-Silesian Medical Centre of the Medical University of Silesia, Katowice, Poland; ^4^ Medical University of Warsaw, Warsaw, Masovian, Poland; ^5^ MindHealth, Warsaw, Poland

**Keywords:** ketamine, esketamine, depression, treatment resistant depression (TRD), borderline personality disorder, ketamine-assisted psychotherapy (KAT)

In the published article, there was an error in the affiliations for authors Magdalena Więdłocha, Piotr Marcinowicz and Agata Szulc. Two affiliations were not disclosed. Authors MW and PM were employed by KeyClinic. Author AS was employed by Mindhealth.

In the published article, there was an error in the **Conflict of Interest** statement, which previously stated:

“The authors declare that the research was conducted in the absence of any commercial or financial relationships that could be construed as a potential conflict of interest.”

The corrected statement appears below:

“Authors MW and PM were employed by the company KeyClinic, a commercial mental health center which provides ketamine treatment, as one of many services. Author AS was employed by the company MindHealth, a commercial psychiatric center. AS was also a member of Janssen Cilag Advisory Board and gave lectures for Janssen Cilag.

The remaining authors declare that the research was conducted in the absence of any commercial or financial relationships that could be construed as a potential conflict of interest.”

The authors apologize for these errors and state that this does not change the scientific conclusions of the article in any way. The original article has been updated.

